# Oncologic Outcomes for Different Axillary Staging Techniques in Patients with Nodal-Positive Breast Cancer Undergoing Neoadjuvant Systematic Treatment: A Cancer Registry Study

**DOI:** 10.1245/s10434-024-15292-y

**Published:** 2024-05-06

**Authors:** André Pfob, Daria B. Kokh, Irina Surovtsova, Fabian Riedel, Philipp Morakis, Joerg Heil

**Affiliations:** 1 Department of Obstetrics and Gynecology, Heidelberg University Hospital, Heidelberg, Germany; 2 National Center for Tumor Diseases (NCT), German Cancer Research Center (DKFZ), Heidelberg, Germany; 3 Klinische Landesregisterstelle, Krebsregister Baden-Württemberg, Stuttgart, Germany; 4 Breast Centre Heidelberg, Klinik St. Elisabeth, Heidelberg, Germany

**Keywords:** Breast cancer, Nodal positive, Axillary staging, Cancer registry

## Abstract

**Background:**

Targeted approaches such as targeted axillary dissection (TAD) or sentinel lymph node biopsy (SLNB) showed false-negative rates of < 10% compared with axillary lymph node dissection (ALND) in patients with nodal-positive breast cancer undergoing neoadjuvant systemic treatment (NAST). We aimed to evaluate real-world oncologic outcomes for different axillary staging techniques.

**Methods:**

We identified nodal-positive breast cancer patients undergoing NAST from 2016 to 2021 from the state cancer registry of Baden-Wuerttemberg, Germany. Invasive disease-free survival (iDFS) was assessed using Kaplan–Meier statistics and multivariate Cox regression models (adjusted for age, ypN stage, ypT stage, and tumor biologic subtype).

**Results:**

A total of 2698 patients with a median follow-up of 24.7 months were identified: 2204 underwent ALND, 460 underwent SLNB (255 with ≥ 3 sentinel lymph nodes [SLNs] removed, 205 with 1–2 SLNs removed), and 34 underwent TAD. iDFS 3 years after surgery was 69.7% (ALND), 76.6% (SLNB with ≥ 3 SLNs removed), 76.7% (SLNB with < 3 SLNs removed), and 78.7% (TAD). Multivariate Cox regression analysis showed no significant influence of different axillary staging techniques on iDFS (hazard ratio [HR] for SLNB with < 3 SLNs removed 0.96, 95% confidence interval [CI] 0.62–1.50; HR for SLNB with ≥ 3 SLNs removed 0.86, 95% CI 0.56–1.3; HR for TAD 0.23, 95% CI 0.03–1.64; ALND reference), and for ypN+ (HR 1.92, 95% CI 1.49–2.49), triple-negative breast cancer (HR 2.35, 95% CI 1.80–3.06), and ypT3-4 (HR 2.93, 95% CI 2.02–4.24).

**Conclusion:**

These real-world data provide evidence that patient selection for de-escalated axillary surgery for patients with nodal-positive breast cancer undergoing NAST was successfully adopted and no early alarm signals of iDFS detriment were detected.

**Electronic supplementary material:**

The online version of this article (10.1245/s10434-024-15292-y) contains supplementary material, which is available to authorized users.

Therapeutic breast cancer management has undergone several paradigm shifts over the past decades. Radical surgery, including mastectomy and axillary lymph node dissection (ALND), used to be the standard of care during the Halstedian paradigm. With the introduction of modern, multimodality treatment (surgery, systemic treatment, radiotherapy), more tailored and less invasive surgical interventions such as breast-conserving surgery and sentinel lymph node biopsy (SLNB) were established. These methods are based on clinical trials that demonstrate non-inferior oncologic outcomes.^1–4^ Breast-conserving surgery and SLNB were initially established in the adjuvant setting and also subsequently in the neoadjuvant setting.

However, an important knowledge gap remains in the optimal axillary staging technique for patients with nodal-positive breast cancer undergoing neoadjuvant systemic treatment (NAST). Several diagnostic studies compared the diagnostic performance of SLNB with ALND and demonstrated that SLNB has a high risk of leaving tumor behind (false-negative rate > 10%).^5–7^ More recently, targeted approaches such as targeted axillary dissection (TAD) or SLNB with ≥ 3 sentinel lymph nodes (SLNs) removed showed missed cancer rates of < 10% compared with ALND.^7–9^ Based on these diagnostic results, international guidelines have permitted the use of such targeted axillary staging techniques instead of ALND;^10,11^ however, to date, there is no evidence on actual oncologic outcomes for de-escalated axillary staging following NAST.

In this study, we evaluated oncologic outcomes for different axillary staging techniques in nodal-positive breast cancer patients undergoing NAST. We utilized data reported to the cancer register of Baden-Wuerttemberg and thus represented routine clinical practice in a real-world patient population.

## Methods

### Patient Selection

Patient records were obtained from the clinical Cancer Registry Database (Klinisches Landeskrebsregister [KLR]) of the German Federal State Baden-Wuerttemberg (BW), with a population of over 11 million in 2019. The KLR BW collects standardized clinical, diagnostic, treatment, and follow-up information for all patients who are diagnosed with cancer in the BW state. The standardized data transfer from the treating cancer centers to the cancer registry includes patient age, estrogen receptor (ER) status, progesterone receptor (PR) status, HER2 neu receptor status, grading, tumor biology, cTNM stage, pTNM stage, lymph nodes removed, lymph nodes with cancer, type of breast surgery (mastectomy vs. breast-conserving), and type of axillary staging (SLNB vs. ALND) and follow-up (recurrences, remission, death, etc.).

### Patient Selection

**Fig. 1:**
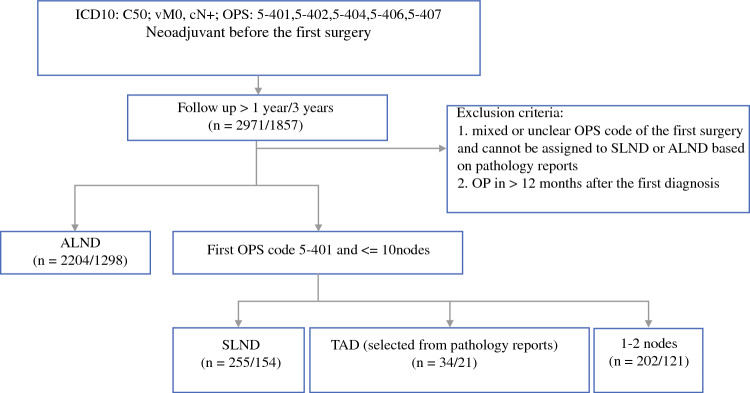
Study flow chart. *ALND* axillary lymph node dissection, *ICD10* International Classification of Diseases, Tenth Revision, *OPS* operation and procedure, *SLND* sentinel lymph node dissection, *TAD* targeted axillary dissection

The inclusion criteria for selecting patients were (1) malignant neoplasm of the breast (International Classification of Diseases, Tenth Revision [ICD-10] code C50) diagnosed between 2016 and 2021; (2) TNM clinical classification of the lymph node and metastases were cN+ and cM0, respectively; (3) reported neoadjuvant therapy before the first surgery; (4) standardized surgical procedure codes (operation and procedure [OPS] codes) of first surgery included either an ALND or SLNB code (i.e. OPS code is either from 5-401, 5-402, 5-404, 5-406, 5-407); and (5) follow-up was available for at least 1 year. The timeframe was chosen with regard to the introduction of TAD in 2016^8^ and at least 1-year of follow-up data. Patients with metastatic disease were not included in the analysis. All selection procedure steps are illustrated in Fig. 1.

To collect information regarding the diagnosis and therapy, the standardized database records, as well as pathology reports, were considered. Information retrieved from the database included patient age, ER and PR status, HER2 neu receptor status, grading, tumor biology, cTNM stage, pTNM stage, and type of (axillary) surgery (OPS code). Due to its novelty, TAD is not yet part of the standardized data capturing and therefore was identified based on the pathology report text (see below). Pathology reports of the patients selected by diagnosis year, ICD-10, and OPS code were automatically scanned using an in-house text analysis program (overall, 22,805 pathology reports). The number of removed lymph nodes, pTNM code, and the references to NAST, sentinel lymph node dissection, or marking were searched, extracted, and analyzed in addition to the available records in the database. Cases with unknown or ambiguous information in any of these variables were excluded.

The patient groups were built using the following multistep procedure:

1. First, patients who had ALND as the first OPS code (OPS codes 5-402, 5-404, 5-406, 5-407) or those who recorded SLNB as the first OPS code (OPS code 5-401), but in whom the number of lymph nodes removed was above 10, were assigned to the ALND group.
2. We then identified patients with a first OPS code of 5-401 (excision of single lymph nodes) and < 11 SLNs removed, and with available full-text pathology reports (for 339 patients in total), which allowed for text-based analysis as to whether TAD was performed (removal of the clipped lymph node plus SLNB).
3. Preliminary selection by text mining; 171 pathology reports were then manually inspected, and the TAD group was extracted based on analysis of the pathology reports (removal of the clipped lymph node plus SLNB).
4. The remaining patients with a first OPS code of 5-401 and < 11 SLNs removed (including those without a pathology report available) were assigned to either the SLNB with < 3 SLNs removed group or the SLNB with ≥ 3 SLNs removed group.

### Statistical Analysis

Invasive disease-free survival (iDFS) was assessed using Kaplan–Meier statistics and multivariate Cox regression models (adjusted for age, ypN stage, ypT stage, and tumor biologic subtype). Time to event was defined as the time from surgery to an event, and events were defined as either local recurrence, distant recurrence, or death. Univariate and multivariate Cox proportional hazard models were used to evaluate the prognostic value of the different axillary staging techniques on the risk of experiencing an event. Hazard ratios (HRs) per unit score are reported. Kaplan–Meier estimators were used to obtain the iDFS rates after 2 and 4 years of follow-up. Time to event was compared using log-rank tests.

### Ethics

Ethical review and approval were waived for this study due to the de-identified information of patients included in the BW database.

## Results

### Flow of Participants

A total of 3307 patients with cN+, cM0 breast cancer undergoing NAST and axillary surgery from 2016 to 2021 were included, of whom 2971 had at least 1 year of follow-up and 2698 had available information regarding axillary staging techniques (see also Fig. 1).

### Patient Demographic and Clinical Information

Table 1 illustrates patient demographic and clinical information. Among the 2698 patients analyzed, median patient age was 59.6 years (standard deviation [SD] 14.4). With respect to tumor stage, 36.8% (605/1643) accounted for pT0 stage and 50.8% (1121/2205) accounted for pN0 stage. Tumor biology was hormone receptor (HR)-positive/HER2-negative in 40.2% (1043/2594) of cases, HR-positive/HER2-positive in 19.8% (513/2594), HR-negative/HER2-positive in 10.3% (266/2594), triple-negative in 16.7% (433/2594), and changing receptor status in 13.1% (339/2594). Axillary staging technique was ALND in 81.7% (2204/2698) of patients, SLNB with ≥ 3 SLNs removed in 9.5% (255/2698), SLNB with < 3 SLNs removed in 7.6% (205/2698), and TAD in 1.3% (34/2698). Distribution of baseline clinical and patient characteristics for the different axillary staging techniques is also listed in Table 1. There were significant differences for c/ypT stage, c/ypN stage, tumor biologic subtype, type of neoadjuvant treatment, and adjuvant radiotherapy among the different axillary staging groups, with higher tumor stages in the ALND group. Notably, there were no significant differences for irradiation volumes among the ALND patients (53.6, SD 6.7), SLNB patients with ≥ 3 SLNs removed (55.3, SD 5.9), SLNB patients with < 3 SLNs removed (53.8, 6.1), and TAD patients (50.4, SD 10.0).

**Table 1:** Baseline clinical and patient characteristics

	Overall	ALND	SLNB (≥ 3 SLNs removed)	SLNB (< 3 SLNs removed)	TAD	*p*-value
Axillary staging techniques	2698 (100)	2204 (81.7)	255 (9.5)	205 (7.6)	34 (1.3)	< 0.001
Patient age, years [median (SD)]	60.4 (14.4)	60.8 (14.5)	59.1 (13.8)	59.2 (14.1)	54.2 (10.5)	0.001
cT stage						< 0.001
cT0	9 (0.3)	8 (0.4)	0	1 (0.0)	0	
cT1–2	1991 (74.8)	1584 (72.7)	210 (83.7)	166 (83.4)	31 (91.2)	
cT3–4	662 (24.9)	586 (26.9)	41 (16.3)	32 (16.1)	3 (8.8)	
cN stage						< 0.001
cN1	2322 (86.7)	1867 (85.1)	236 (94.0)	187 (94.4)	32 (94.1)	
cN2	244 (9.1)	226 (10.3)	9 (3.6)	7 (3.5)	2 (5.9)	
cN3	111 (4.1)	101 (4.6)	6 (2.4)	4 (2.0)	0	
pT stage						< 0.001
pT0	605 (36.8)	466 (34.4)	65 (46.4)	64 (50.0)	10 (52.6)	
pT1–2	888 (54.0)	747 (55.1)	71 (50.7)	63 (49.2)	7 (36.8)	
pT3–4	150 (9.1)	143 (10.5)	4 (2.9)	1 (0.8)	2 (10.5)	
pN stage						< 0.001
pN0	1121 (50.8)	825 (46.3)	141 (66.2)	139 (74.7)	16 (69.6)	
pN+	1084 (49.2)	958 (53.7)	72 (33.8)	47 (25.3)	7 (30.4)	
Tumor biologic subtype						0.0053
HR-positive/HER2-negative	1043 (40.2)	888 (41.9)	94 (38.5)	47 (23.7)	14 (41.2)	
HR-positive/HER2-positive	513 (19.8)	404 (19.1)	53 (21.7)	49 (24.7)	7 (20.6)	
HR-negative/HER2-positive	266 (10.3)	215 (10.2)	21 (8.6)	24 (12.1)	6 (17.6)	
TNBC	433 (16.7)	339 (16.0)	44 (18.0)	46 (23.2)	4 (11.8)	
Changing receptor status	339 (13.1)	272 (12.8)	32 (13.1)	32 (16.2)	3 (8.8)	
Neoadjuvant treatment						< 0.001
Chemotherapy + anti HER2	281 (13.1)	220 (12.5)	22 (10.8)	29 (18.2)	10 (33.3)	
Chemotherapy + immunotherapy	11 (0.5)	10 (0.6)	0 (0.0)	0 (0.0)	1 (3.3)	
Chemotherapy	1854 (86.4)	1524 (86.9)	181 (89.2)	130 (81.8)	19 (63.3)	
Radiotherapy						
Whole breast	935 (50.2)	739 (47.6)	105 (64.4)	85 (65.4)	6 (42.9)	
Partial breast	342 (18.4)	273 (17.6)	30 (18.4)	33 (25.4)	6 (42.9)	
Thoracic wall	584 (31.4)	542 (34.9)	12 (9.2)	28 (17.1)	2 (14.3)	
Radiotherapy volume [median (SD)]	53.8 (6.5)	53.6 (6.7)	55.3 (5.9)	53.8 (6.1)	50.4 (10.0)	0.76

Data are expressed as *n* (%) unless otherwise specified

*ALND* axillary lymph node dissection, *HER2* human epidermal growth factor receptor, *HR* hormone receptor, *SD* standard deviation, *SLNB* sentinel lymph node biopsy, *SLNs* sentinel lymph nodes, *TNBC* triple-negative breast cancer

### Oncologic Outcomes for Different Axillary Staging Techniques

**Fig. 2:**
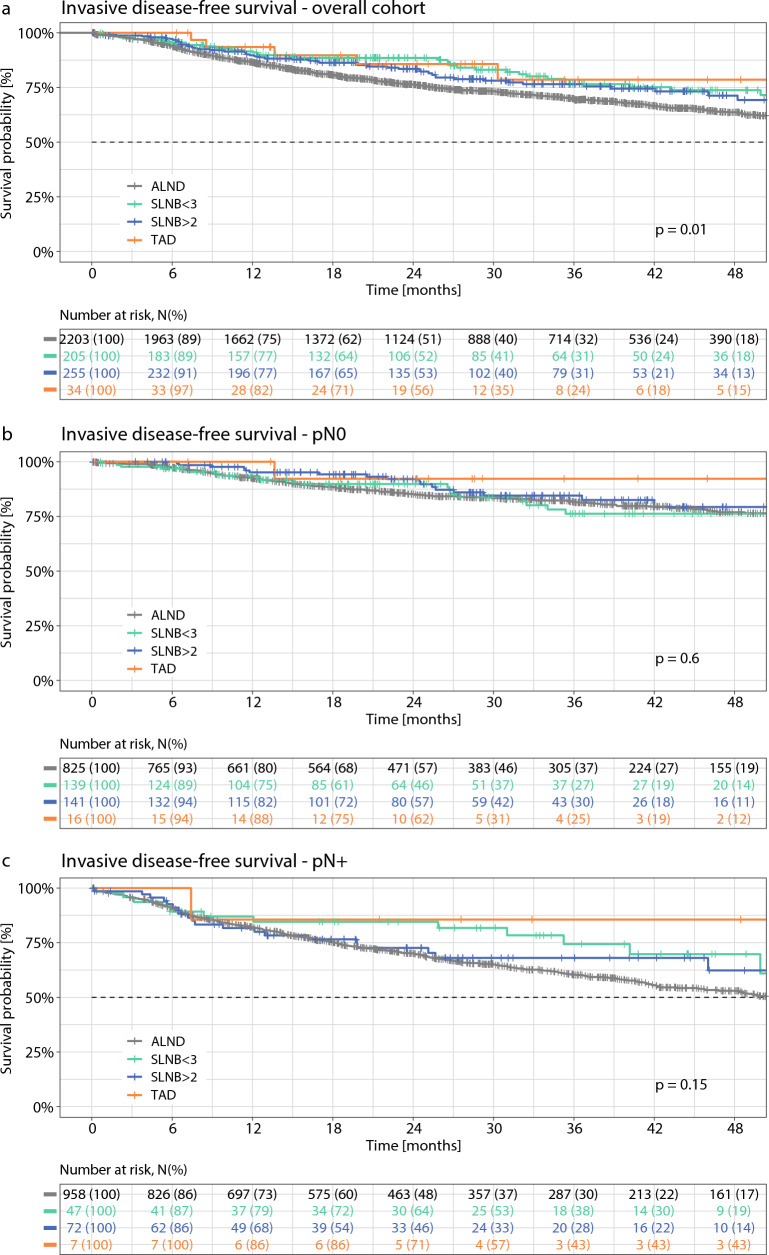

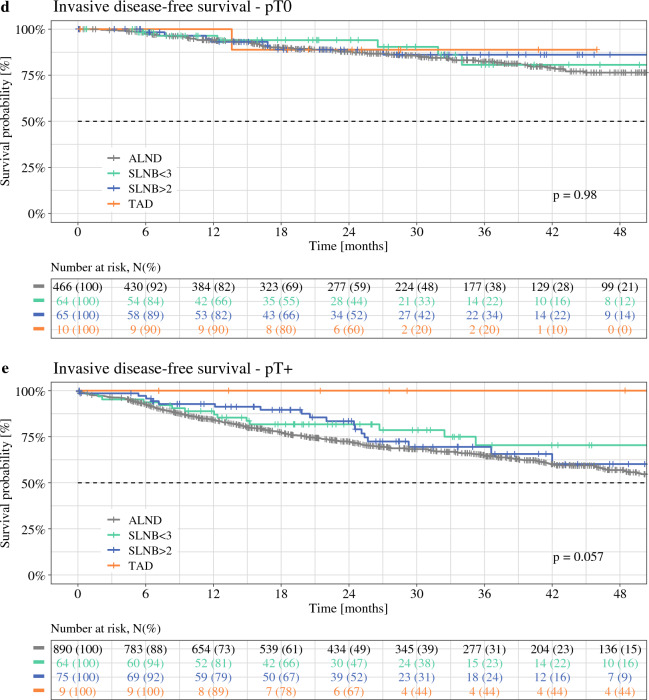
Kaplan–Meier plots of invasive disease-free survival for different axillary staging techniques. (**a**) Whole cohort; (**b**) ypN0 stage; (**c**) ypN+ stage; (**d**) ypT0 stage; (**e**) ypT+ stage. *ALND* axillary lymph node dissection, *SLNB* sentinel lymph node biopsy, *TAD* targeted axillary dissection

**Fig. 3:**
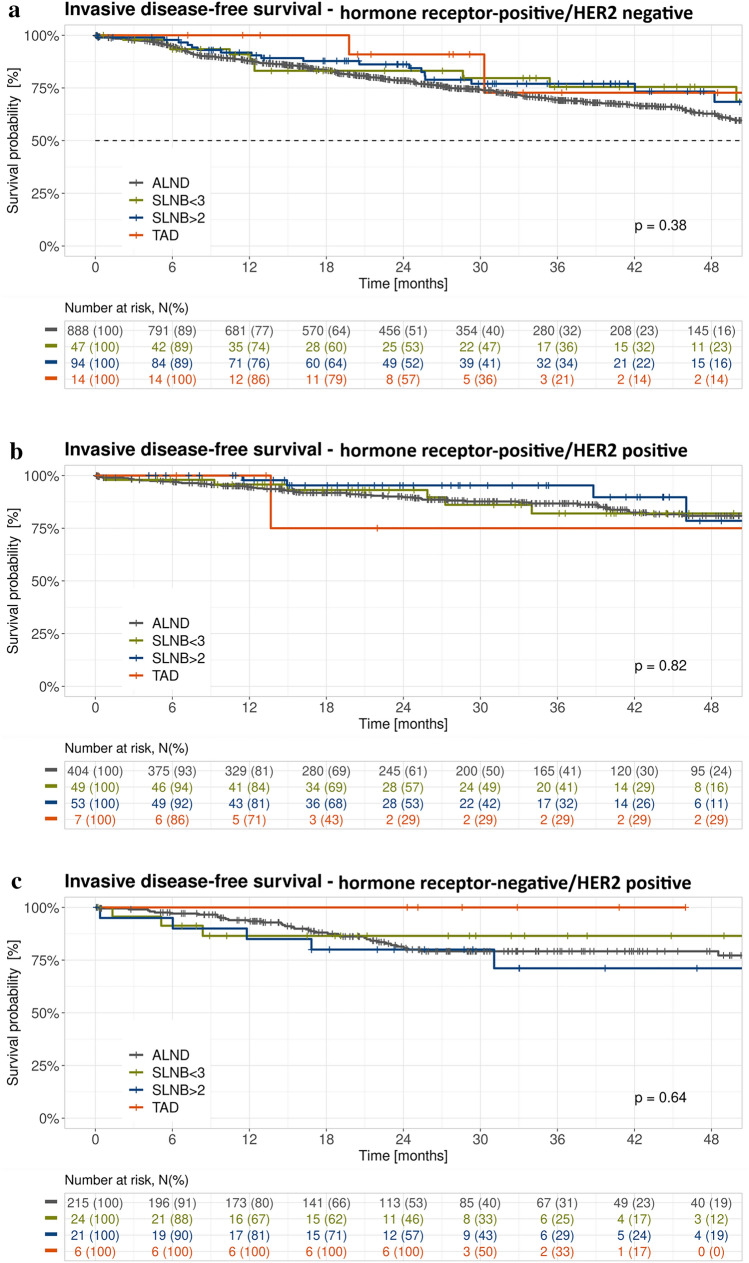

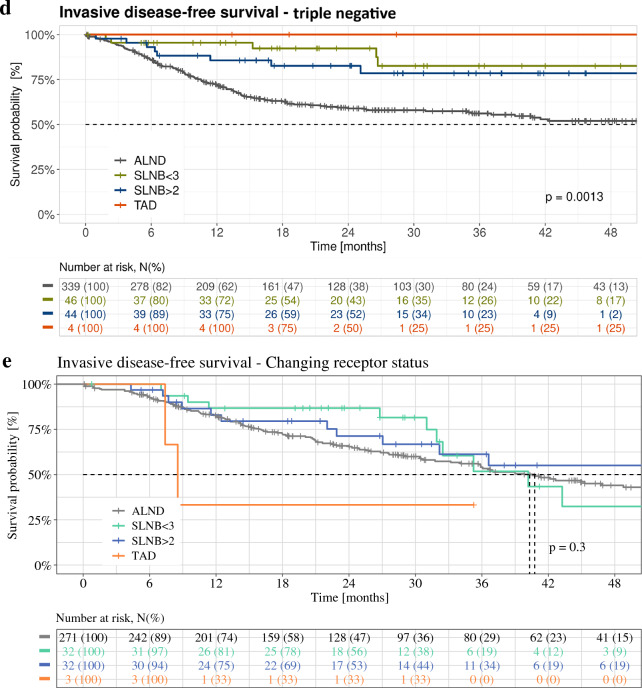
Kaplan–Meier plots of invasive disease-free survival for different axillary staging techniques. (**a**) HR-positive/HER2-negative; (**b**) HR-positive/HER2-positive; (**c**) HR-negative/HER2-positive; (**d**) TNBC; (**e**) changing receptor status. *ALND* axillary lymph node dissection, *HR* hormone receptor, *HER2* human epidermal growth factor receptor 2, *SLNB* sentinel lymph node biopsy, *TAD* targeted axillary dissection, *TNBC* triple-negative breast cancer

Median follow-up was 24.7 months. Kaplan–Meier plots of iDFS for the different axillary staging techniques (ALND, SLNB, and TAD) in the whole cohort and stratified for ypN+ versus ypN0, ypT+ versus ypT0, and tumor biologic subtype are illustrated in Figs. 2 and 3. In the overall cohort, significant differences in iDFS between the ALND, SLNB, and TAD groups were observed (log rank *p* = 0.01): iDFS 3 years after surgery was 69.7% (ALND), 76.6% (SLNB with ≥ 3 SLNs removed), 76.7% (SLNB with < 3 SLNs removed), and 78.7% (TAD).

**Fig. 4:**
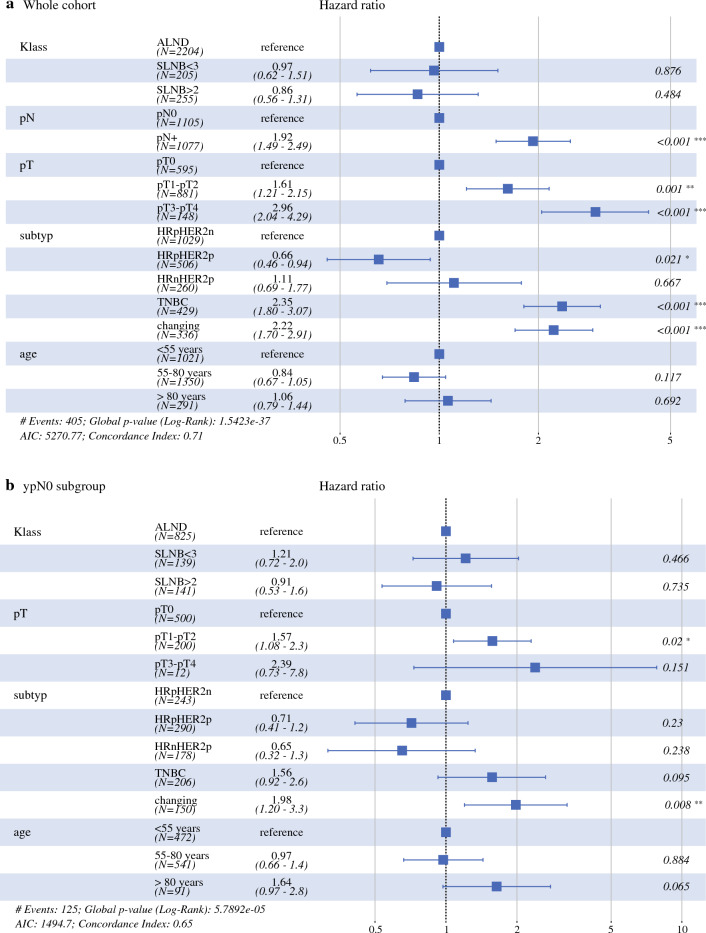
Multivariate Cox regression analysis without the TAD group. (**a**) Whole cohort; (**b**) ypN0 subgroup. *AIC* Akaike information criterion, *ALND* axillary lymph node dissection, *HRn* hormone receptor-negative, *HRp* hormone receptor-positive, *HER2n* human epidermal growth factor receptor 2-negative, *HER2p* human epidermal growth factor receptor 2-positive, *SLNB* sentinel lymph node biopsy, *TAD* targeted axillary dissection, *TNBC* triple-negative breast cancer

Multivariate Cox regression analysis, including axillary staging technique (without TAD due to the low sample size), age, pTNM stage, and tumor biologic subtype (Fig. 4), showed no significant influence of different axillary staging techniques on iDFS: HR 0.86 (95% confidence interval [CI] 0.56–1.31) for SLNB with ≥ 3 SLNs removed; HR 0.97 (95% CI 0.62–1.51) for SLNB with <3 SLNs removed; ALND (reference). iDFS was significantly influenced by ypN+ status (HR 1.92, 95% CI 1.49–2.49, *p* < 0.001, compared with ypN0), ypT+ status (HR 1.61, 95% CI 1.21–2.15, *p *< 0.001 for ypT1-2; and HR 2.96, 95% CI 2.04–4.29, *p* < 0.001 for ypT3-4, compared with ypT0), and receptor status (HR 2.35, 95% CI 1.80–3.07, *p *< 0.001 for triple-negative breast cancer (TNBC); and HR 2.22, 95% CI 1.70–2.91, *p *< 0.001 for changing receptor status, compared with HR-positive/HER2-negative). Also in the subgroup of patients with ypN0 status (Fig. 4b), no significant influence of different axillary staging techniques on iDFS was observed: HR 0.91 (95% CI 0.53–1.60) for SLNB with ≥3 SLNs removed; HR 1.21 (95% CI 0.72–2.00) for SLNB with <3 SLNs removed; ALND (reference).

If TAD was included in the multivariate Cox regression analysis, it resulted in an HR of 0.23 (95% CI 0.032–1.64), which should however be interpreted with caution considering the low sample size (Supplemental Fig. 1).

### Subgroup Analysis

When stratified for ypN and ypT status (Fig. 2) as well as receptor status (Fig. 3), differences in iDFS between the different axillary staging techniques were descriptively larger in patients with ypN+ versus ypN0 disease (Fig. 2b, c), in patients with ypT+ versus ypT0 disease (Fig. 2d, e), and in patients with TNBC (Fig. 3d).

**Fig. 5:**
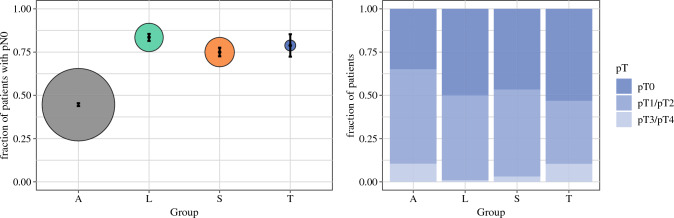
Fraction of patients with residual disease after surgery. Groups A, L, S, and T denote the ALND, SLNB with < 3 SLNs removed, SLNB with ≥3 SLNs removed, and TAD groups, respectively. The bootstrapping procedure was used for computation of mean and standard deviation values in each group. *ALND* axillary lymph node dissection, *SLNB* sentinel lymph node biopsy

Figure 5 illustrates the fractions of patients with ypN0 versus ypN+ and ypT0 versus ypT+ status for the different axillary staging techniques in patients were both pieces of information were available (*n* = 1612): The proportion of patients with pN0 status was significantly lower in the ALND group compared with the SLNB with ≥ 3 SLNs removed, SLNB with < 3 SLNs removed, and TAD groups (46.3% vs. 66.2%, 74.7%, and 69.5%, respectively).

## Discussion

In this study, we evaluated oncologic outcomes for different axillary staging techniques in nodal-positive breast cancer patients undergoing NAST, specifically for ALND, SLNB (≥ 3 and < 3 SLNs removed), and TAD. After a median follow-up of 24.7 months, multivariate Cox regression analysis showed no significant influence of different axillary staging techniques on iDFS in the overall cohort (Fig. 4a). Also in the subgroup of ypN0 patients, where de-escalated axillary surgery is known to occasionally miss residual cancer, no significant influence of different axillary staging techniques on iDFS was observed (Fig. 4b). These data provide evidence that patient selection for de-escalated axillary surgery (in combination with radiotherapy) was successfully adopted in a real-world population sample and no early alarm signals of survival detriment were detected.

We have learned in the past decades that most breast cancer patients can be spared radical surgery in times of modern multimodality treatment. At first, clinical trials demonstrated equivalent survival for breast-conserving therapy (BCT, breast-conserving surgery and radiotherapy) compared with mastectomy in the early 2000s.^10,11^ Later, clinical trials demonstrated equivalent survival of SLNB compared with ALND in the adjuvant setting for cN0 patients, despite leaving some tumor behind (about 10% of missed lymph node metastasis for SLNB compared with ALND).^12,13^ From these trials, the assumption arose that a false-negative rate of 10% would not translate into impaired oncologic outcomes. Consequently, as subsequent trials showed false-negative rates of >10% for SLNB in cN+ patients undergoing NAST, ALND remained the standard of care for these patients. Recent improvements in less invasive targeted axillary staging techniques showed improved false-negative rates compared with ALND: about 2% for TAD (removal of clipped node plus SLNB)^8,9,14^ and about 8% for SLNB with ≥ 3 SLNs removed.^14^ Notably, based on these diagnostic findings, international guidelines now allow the use of such targeted axillary staging for cN+ patients undergoing NAST despite missing survival data. Another recent study evaluated 3-year oncologic outcomes of 199 patients undergoing TAD (*n* = 119) versus TAD with ALND (80). After 3 years of follow-up, iDFS was 82.4% (95% CI 71.5–89.4) in the TAD + ALND group, and 91.2% (95% CI 84.2–95.1) in the TAD group (*p* = 0.04); axillary recurrence occurred in 1.4% (95% CI 0–54.8) and 1.8% (95% CI 0–36.4), respectively (*p* = 0.56). TAD was not associated with an increased risk of recurrence (HR 0.83, 95% CI 0.34–2.05; *p* = 0.69) or death (HR 1.07, 95% CI 0.31–3.70; *p* = 0.91) in the adjusted multivariate Cox regression.^15^ Our analyses provides additional information, especially with respect to the clinically relevant subgroup of patients with ypN0 disease, where SLNB or TAD is known to occasionally miss residual disease (false negative rate, see above). Also in the subgroup of patients with ypN0 status (Fig. 4b), no significant influence of different axillary staging techniques on iDFS was observed, with an HR of 0.91 (95% CI 0.53–1.60) for SLNB with ≥ 3 SLNs removed and an HR of 1.21 (95% CI 0.72–2.00) for SLNB with < 3 SLNs removed. Notably, our registry analysis also provides insights into irradiation practices in case of de-escalated surgical axillary staging: no significant differences were observed for irradiation volumes among the ALND (53.6, SD 6.7), SLNB with ≥ 3 SLNs removed (55.3, SD 5.9), SLNB with < 3 SLNs removed (53.8, 6.1), and TAD (50.4, SD 10.0) groups. Although we acknowledge the small sample size in some groups, there does not seem to be a trend for more irradiation in the case of de-escalated surgical staging. Thus, the results of our analysis provide additional information regarding the oncologic outcomes for SLNB, and add to the available survival outcomes for different axillary staging techniques in nodal-positive breast cancer patients undergoing NAST.

Tailored oncologic breast and axillary surgery may not only result in equivalent survival but may actually improve survival. A recent meta-analysis comparing BCT with mastectomy in times of modern multimodality (14 studies from 1980 to 2014, *n* = 19,819) suggests that BCT is actually associated with improved oncologic outcomes: all-cause mortality in favor of BCT (HR 0.78, 95% CI 0.69–0.89; *p* < 0.001), locoregional recurrence in favor of BCT (HR 0.64, 95% CI 0.48–0.85; *p* = 0.002), and distant recurrence in favor of BCT (HR 0.70, 95% CI 0.530.94; *p* = 0.02).^3^ Mechanisms of action include reduction of rare but severe surgical complications such as thrombosis and sepsis. In our study, significant differences in iDFS between the ALND, SLNB, and TAD groups were observed in favor of the targeted approaches (log rank *p* = 0.01), which may however reflect differences in tumor stages among the axillary staging groups. For example, the proportion of patients with ypN+ stage within these four groups was 53.5%, 33.8%, 25.2%, and 30.4%, respectively (*p *< 0.001). When adjusting for ypT/ ypN stages, age, and tumor biologic subtype, no significant influence on iDFS for the different axillary staging techniques was observed. Further evaluation in larger (registry) trials with longer follow-up seems warranted.

The interactions between axillary staging and adjuvant systemic therapy are an emerging topic of high relevance. For example, pN status is frequently used to identify high-risk patients eligible for escalated adjuvant treatment (abemaciclib for HR-positive/HER2-negative patients with pN2 status or pN1 status with additional risk factors, trastuzumab emtansine (T-DM1) for HER2-positive patients with residual disease, and capecitabine for TNBC patients with residual disease).^16–18^ It should be noted that the differences in distribution of pN status between different axillary staging techniques observed in our study may not be completely related to the actual disease stage. A higher proportion of patients with pN+ status in the ALND group may also reflect the fact that we actually leave some tumor behind in the axilla when using targeted techniques so that the proportion of patients with pN+ stage is systematically lower within these populations. Indeed, the American College of Surgeons (ACOSOG) Z0011 trial showed that almost 14% of patients who underwent SLNB with 1–2 positive SLNs had ≥4 positive nodes after undergoing subsequent ALND.^4^ Thus, future research may develop new risk scores to identify patients with (high) nodal disease burden for accurate indications of escalated adjuvant systemic treatment in times of de-escalated axillary surgery.

A critical discussion focuses around the role of clinical lymph node assessment after NAST. Current national guidelines allow the use of targeted axillary staging techniques (SLNB, TAD) for patients with nodal-positive disease before NAST (cN+) who convert to nodal-negative disease (ycN0) on imaging, whereas patients who remain nodal positive (ycN+) on imaging are recommended to undergo ALND.^10,11^ In the present analysis, patients with cN+ disease were included but ycN status is unknown as this information is not routinely collected by the cancer registry. There is however an ongoing discussion about the usefulness of axillary response assessment by imaging after NAST. Axillary ultrasound, which is most commonly used to assess response of the axillary lymph nodes, shows limited diagnostic accuracy to determine the ycN status; among patients with negative nodes on ultrasound after NAST (ycN0), about 50% present with residual axillary disease in the surgical specimen.^19^ Taking ycN0 status as a prerequisite for targeted axillary staging techniques may thus be inadequate and ineffective. Future research may focus on this area and provide more in-depth comparisons of oncologic outcomes for patients undergoing targeted axillary staging with ycN+ status.

Some limitations of the present analysis must be kept in mind. First, the sample size of the TAD group was small, which is why this group was not included in the multivariate Cox regression analysis. Moreover, a median follow-up of 24.7 months may enable first conclusions with respect to local recurrence events, but does not suffice to conclude safe long-term overall or distant DFS. Future studies with longer-term follow-up are welcomed to fully inform this discussion. Second, as TAD is relatively new, it is not routinely captured by the cancer registry. We identified patients with available full-text pathology reports, which allowed for text-based analysis as to whether TAD was performed (removal of clipped lymph node plus SLNB); however, this approach may have led to selection bias).

## Conclusion

These real-world data provide evidence that patient selection for de-escalated axillary surgery (such as TAD or SLNB instead of ALND) in combination with radiotherapy for patients with nodal-positive breast cancer undergoing NAST was successfully adopted in a real-world population sample and no early alarm signals of iDFS detriment were detected. Future studies with longer-term follow-up are encouraged to fully inform this discussion.

### Electronic supplementary material

Below is the link to the electronic supplementary material.

Supplementary file 1 (DOCX 242 kb)

## Data Availability

Will individual participant data be available (including data dictionaries)?: No. What data in particular will be shared?: No. What other documents will be available?: Study protocol. When will data be available (start and end dates)?: No. With whom?: No. For what types of analyses?: No. By what mechanism will data be made available?: No
