# Supplementary figures and images for: Oncologic Outcomes for Different Axillary Staging Techniques in Patients with Nodal-Positive Breast Cancer Undergoing Neoadjuvant Systematic Treatment: A Cancer Registry Study
Source: Ann Surg Oncol. 2024 May 6;31(7):4381–92. doi: 10.1245/s10434-024-15292-y (PMC11164833; doi:10.1245/s10434-024-15292-y)

Supplemental Figure 1: Multivariate Cox Regression Analysis with TAD group


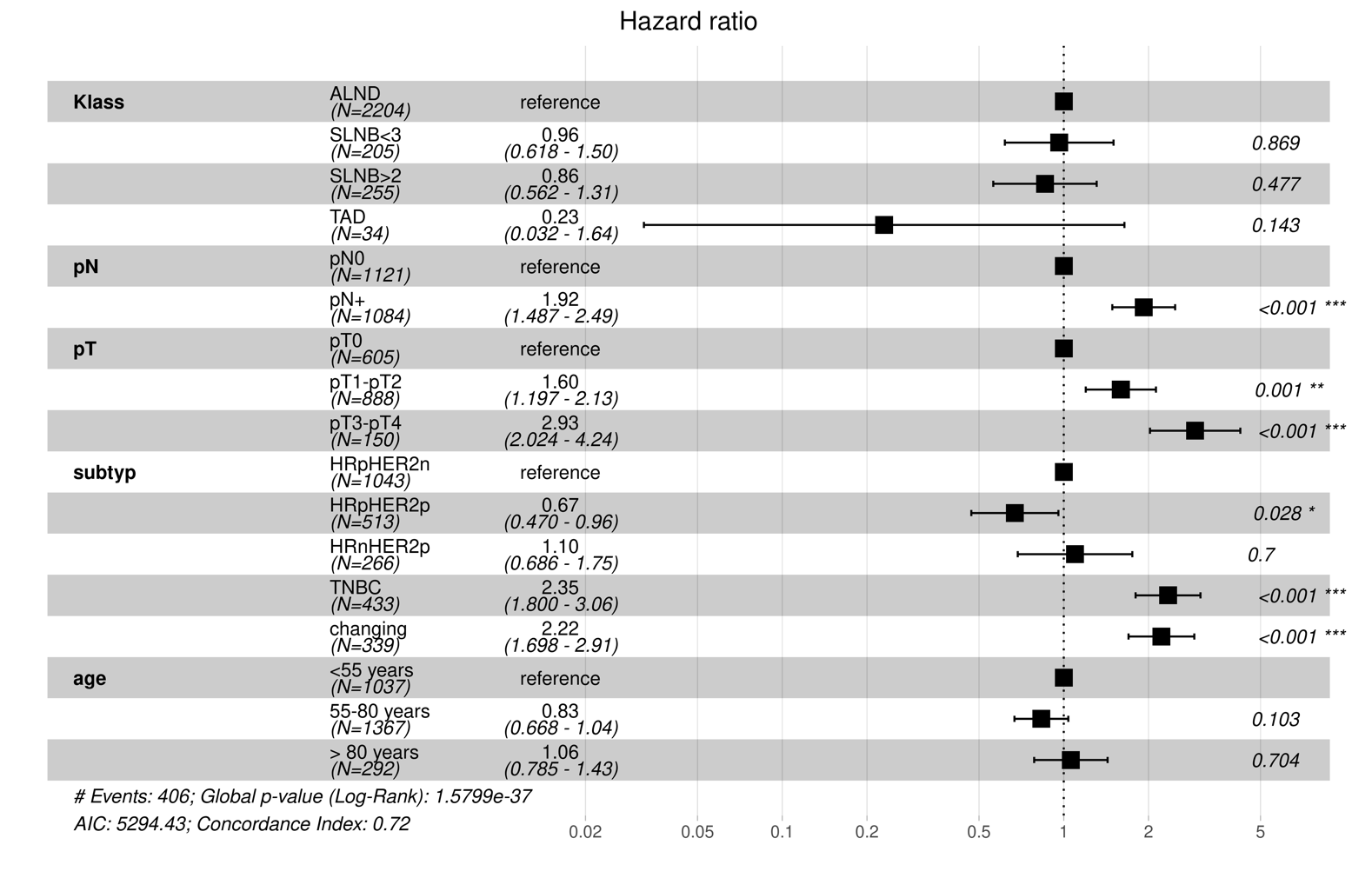

Supplement: Supplementary file 1 — (DOCX 242 kb) [file 10434_2024_15292_MOESM1_ESM.docx]
